# The Clinical Application of Ingluvin

**Published:** 1893-09

**Authors:** John V. Shoemaker


### THE CLINICAL APPLICATION OF INGLUVIN.

Ingluvin is the name given to a preparation made from the gizzard of the domestic fowl. It is a yellowish, gray powder, of a faint odor, and almost devoid of taste. It is insoluble in water. Ingluvin is put up by its manufacturers, (Messrs. William R. Warner & Co., of Philadelphia,) in 5-grain tablets. Ingluvin is compatible with alkalies. Its virtues reside in a peculiar bitter principle which enters into its composition. It is prescribed in the same doses and combinations as pepsin. Ingluvin was introduced to the notice of the medical profession about eighteen years ago. It is of special benefit in the relief of sick stomach. This substance may be given with success when vomiting depends upon organic affection of the stomach, as in acute and chronic gastric catarrh and in gastric ulcer. Nausea, due to disease of other abdominal or pelvic viscera, as the liver, kidneys, uterus and ovaries, is likewise relieved by the administration of this remedy. It allays the gastric irritability which accompanies tabes-mesenterica and marasmus. Vomiting produced by over-indulgence in liquor has been subdued by its powers. It has been found of advantage in cases of sea-sickness, and in the relief of the gastric irritability of bottle-fed babes. Its peculiar province, however, is alleviation of the vomiting of pregnancy, in which it approaches the character of a specific. As every one knows, this difficulty is frequently very intractable, and one approved remedy after another may be used without avail. To those who have witnessed repeated failures of medication, Ingluvin can be recommended as one of the most efficient remedies which we possess for the relief of this distressing symptom. Ingluvin is likewise beneficial in dyspepsia, when produced by functional inactivity. It is able to promptly check the diarrhœa which is caused by indigestion. By reason of its influence upon the stomach and bowels, Ingluvin is capable of marked service in cases of cholera infantum and cholera morbus. From the preceding account it will be seen that Ingluvin possesses an exceedingly important sphere of usefulness.

Ten grains I found generally a sufficient dose. In some instances twenty grains were required, while in the milder forms of indigestion a 5-grain tablet, after each meal, accomplished the desired purpose. To infants I gave the remedy in doses of one or two grains.

A series of cases occurring during the past few years in which Ingluvin was administered with benefit has been selected as affording a typical example of the efficacy of Ingluvin. The total number amounted to 49 and a brief history is given of each case. They were classified as follows :—4 cases of cholera morbus ; 8 of infantile diarrhœa ; 9 of diarrhœa in the adult ; 2 of dysenteric diarrhœa ; 1 of acute indigestion ; 3 of dyspepsia ; 2 of dyspepsia with reflex symptoms ; 1 of dyspepsia from uterine disease ; 2 of flatulent dyspepsia ; 1 of nervous dyspepsia ; 2 of gastralgia ; 2 of colic ; 4 of gastric and gastro-intestinal catarrh ; 1 of gastric ulcer ; 1 of vomiting caused by alcoholism ; 6 of vomiting of pregnancy.—*Abstract of a paper by John V. Shoemaker, A. M., M. D., in the Medical Bulletin for June*, 1893.

